# Spatial-Temporal Study of Rab1b Dynamics and Function at the ER-Golgi Interface

**DOI:** 10.1371/journal.pone.0160838

**Published:** 2016-08-08

**Authors:** Hernán Martinez, Iris A. García, Luciana Sampieri, Cecilia Alvarez

**Affiliations:** Centro de Investigaciones en Bioquímica Clínica e Inmunología (CIBICI-CONICET), Departamento de Bioquímica Clínica, Facultad de Ciencias Químicas, Universidad Nacional de Córdoba, Córdoba 5000, Argentina; Institut Jacque Monod, Centre National de la Recherche Scientifique, FRANCE

## Abstract

The GTPase Rab1b is involved in ER to Golgi transport, with multiple Rab1b effectors (located at ERES, VTCs and the Golgi complex) being required for its function. In this study, we performed live-cell dual-expression studies to analyze the dynamics of Rab1b and some effectors located at the ERES-Golgi interface. Rab1b occupied widely distributed mobile punctate and tubular structures, displaying a transient overlaps with its effectors and showing that these overlaps occurred at the same time in spatially distinct steps of ER to Golgi transport. In addition, we assessed Rab1b dynamics during cargo sorting by analyzing the concentration at ERES of a Golgi protein (SialT2-CFP) during Brefeldin A washout (BFA WO). Rab1b was associated to most of the ERES structures, but at different times during BFA WO, and recurrently SialT2-CFP was sorted in the ERES-Rab1b positive structures. Furthermore, we reveal for first time that Rab1b localization time at ERES depended on GBF1, a Rab1b effector that acts as the guanine nucleotide exchange factor of Arf1, and that Rab1b membrane association/dissociation dynamics at ERES was dependent on the GBF1 membrane association and activity, which strongly suggests that GBF1 activity modulates Rab1b membrane cycling dynamic.

## Introduction

Most of the secretory and plasma membrane proteins and other proteins located in compartments that participate in membrane traffic are synthesized in the ER and selectively concentrated in specialized ER domains, known as ER exit sites (ERES), before being exported to their final destinations. Vesicles loaded with proteins destined to be exported (named cargo proteins) bud from ERES and fuse with each other to form the ER-Golgi intermediate compartment (ERGIC, [1]) that comprise clusters of vesicular and tubular shaped membranes (called VTCs). VTCs accumulate in the peri-Golgi region and, once there, form a more extensive array of tubules, sometimes referred to as the cis-Golgi network (CGN). Cargo concentration at ERES takes place through the action of the COPII coat, which is composed of two multimeric protein complexes (Sec23-Sec24 and Sec13-Sec31). Its formation includes activation of the GTPase Sar1, which recruits the Sec23-Sec24 complex and forms an adaptor layer for the Sec13-Sec31 assembly [2]. ERGIC/VTCs represent the target destination of ER export COPII vesicles and form the intermediate between the endoplasmic reticulum and the Golgi stack, where proteins can either move forward to the Golgi or backward to the ER. The 53 kDa membrane protein ERGIC-53 is the conventional marker that defines the ERGIC compartment. At the ERGIC/VTCs level, the COPII coat is exchanged for the COPI coat, with this exchange being considered a maturation process required for the progress of cargo transport from the ER to the Golgi. However, it is still under discussion whether COPI directly allows anterograde cargo transport to the Golgi or if its participation (during the formation of vesicles required for retrograde transport from the ERGIC back to the ER) is indirect [3]. Recruitment of COPI is mediated by the GTPase Arf1 [4, 5], with the interchange of GDP for GTP on Arfs being mediated by a family of guanine nucleotide exchange factors (GEF, [6]) localized in different structures of the exocytic/endocytic pathways. At the ER—Golgi interface, GBF1 (Golgi-specific brefeldin A resistance factor 1, [7]) is an Arf-GEF that regulates the Arf1-mediated COPI recruitment required for the maturation of VTCs in the proximity of ERES [8–10].

In addition to the COPII and COPI complexes, a considerable number of proteins are localized at the ER-Golgi interface and participate in ER to Golgi transport. Among these, the two isoforms of the GTPase Rab1, Rab1a and Rab1b, are involved in ER to Golgi transport [11–13]. Furthermore, some Rab1 interacting proteins, such as the tethering factors p115 [14], GM130 [15, 16], Giantin [17] and Golgin 84 [18], participate at this stage of transport. P115 localizes to ERES, ERGIC/VTCs and the Golgi, with GM130 localizing to the *cis*-Golgi cisternae, while Giantin localizes predominantly to the cisternal rims of the *medial*-Golgi [19]. Rab1 tethers COPII vesicles to the cis-Golgi by interacting with p115 and GM130, and through interaction with p115 and Giantin it mediates the tethering of recycling COPI vesicles to the Golgi [20]. As well as its participation in tethering events, Rab1b regulates COPII dynamics, and is involved in cargo sorting and COPI recruitment, due to its direct interaction with GBF1 [21].

Previous articles from several groups have reported Rab1b direct interaction with proteins localized in ERES, VTCs, and the Golgi complex. However, the extent of Rab1b co-localization with these structures is unknown. In this work we have quantified, for the first time, the co-localization of Rab1b with ERES, ERGIC/VTCs, and Golgi markers in different transport conditions (steady state and after complete Golgi redistribution to the ER). The data reveal that the highest co-localization of Rab1b was with p115 and GM130. Then, also taking into account that Rab1b in its active form (GTP-bound) interacts with effector proteins that mediate membrane traffic and that the GTP-bound form is membrane associated [22], our data suggest that most Rab1b activity takes place in p115 and GM130-labeled structures. Furthermore, we report that Rab1b was concentrated at the ERES during cargo sorting and that the co-localization time of Rab1b with ERES and its cycle of association/dissociation to them was altered when GBF1 activity was inhibited, indicating that Rab1b interactions at the ERES-Golgi interface play mutually supporting roles. Taken together, our results expand the understanding of the spatial-temporal participation of Rab1 during ER to Golgi transport.

## Materials and Methods

### DNA constructs and antibodies

The GFP-Rab1b constructs have been previously described [23], and GBF1-E794K-Myc and GFP-p115 were generously provided by Dr. Elizabeth Sztul (University of Alabama at Birmingham, AL). Full-length Sec24 cloned into the pEYFP-C1 vector (Clontech, Mountain View, CA, USA) have been previously reported [24]. Dr. Hugo Maccioni (Universidad Nacional de Córdoba, Argentina) supplied the SialT2-CFP plasmid. For generation of mCherry-Rab1b, the GFP-Rab1b construct was subcloned from the pEGFP-C1 vector into the BamHI-XbaI restriction sites of the pmCherry-C1 vector (Clontech, Mountain View, CA, USA).

The following antibodies were used: Rabbit polyclonal antibody to GFP, mouse monoclonal antibody to GM130, rabbit polyclonal antibody to EEA1 (Abcam, Cambridge, UK) and rabbit polyclonal antibody to GalNAcT2 (Sigma-Aldrich, St. Louis, MO, USA).

Monoclonal anti-ERGIC53 G1/93 antibody [25] was provided by Dr. Hans-Peter Hauri (University of Basel, Basel, Switzerland). Rabbit polyclonal anti-Rab1b antibody was purchased from Santa Cruz Biotechnology (Santa Cruz Biotechnology, Santa Cruz, CA, USA), with mouse monoclonal antibody to GBF1 and mouse monoclonal anti-Sec31 being obtained from (BD Biosciences, San Jose, CA, USA).

The secondary antibodies goat anti-rabbit Alexa Fluor 488, goat anti-rabbit Alexa Fluor 594, and goat anti-mouse Alexa Fluor 594, were obtained from Molecular Probes (Invitrogen, Carlsbad, CA, USA).

### Cell culture and transient transfections

HeLa cells were grown in DMEM high glucose supplemented with 10% fetal bovine serum (FBS), 100ug/mL penicillin and 100ug/mL Streptomycin. Cells were grown at 37°C in a 5% CO_2_ atmosphere incubator. Transfection was performed with 2 μl Lipofectamine 2000 reagent per μg plasmid, according to the manufacturer’s instructions (Invitrogen, Carlsbad, CA, USA). In some experiments, two plasmids were mixed (1:1 ratio) and then used for transfection. After 24h of transfection, cells were fixed for microscopy images, or processed for *in vivo* studies.

### Immunofluorescence assays and microscopy images

Fixation and staining of cells was performed as previously described [23]. Microscopy images were obtained using the spectral confocal laser scanning microscope Olympus FluoView^™^ FV1000 (Olympus Latin America), equipped with a 60x/1.42 NA oil immersion objective. Alexa Fluor 488 (or GFP) was excited with a 488 nm argon laser, and fluorescence was collected through a bandpass (BP) 505- to 530-nm emission filter. Alexa Fluor 594/555 (or Cherry) was excited with a 543-nm laser, and fluorescence was collected through a BP 593- to 794-nm or BP 565–615 emission filter, with Alexa Fluor 633 being excited with a 633-nm laser and emission being collected through a BP 655–710 nm. Stacks of images (z-stacks) were obtained for two channels with a step-size of 130 nm covering 50 nm per pixel as the lateral resolution. Immunofluorescence images display a representative single slice from each z-stack. The number of slices taken was variable (from 8–15) and based on the fluorescence signal in the cell of interest. All single slices displayed for each fluorescence channel came from the same position of a z-stack used to perform the quantitative co-localization analysis. Moreover, we also checked that the individual Pearson´s correlation coefficient value (PCC, which is explained in the following section) of the displayed slice was close to the average PCC value determined for the analyzed condition. Images were acquired and processed with Olympus FluoView FV10-ASW 3.1 version software, and final image editing carried out using Adobe Photoshop CS.

### Quantitative co-localization analysis

To perform co-localization assays, we used confocal microscopy and acquired z-stacks images from two channels, with quantitative co-localization of punctate structures being performed using Pearson´s correlation coefficient. Deconvolution of confocal images was carried out using Fiji software (http://fiji.sc/Fiji, Biomedical Imaging Group, EPFL). The Point-Spread Function (PSF) Generator was employed to generate 3D models [26] utilizing the vectorial-based model [27]). PSF generation was performed with the following optics parameters (numerical aperture 1.42, refractive index immersion 1.515, lateral and axial resolution, and wavelength 488, 594 or 633, according to the plasmid transfected) and output stack parameters (size NX = 1024/NY = 1024/NZ = variable).

After generating PSF, deconvolution was performed using Deconvolution Lab plugin for Fiji software. Then, the algorithm Tikhonov-Miller was chosen, specifying the regularization parameter (0.05) and number of iterations. The overlap between different proteins was quantified with Fiji software as follows: For each marker protein, the total stacks of the deconvolved image (32-bit) for each channel were used to define a threshold mask that included all of the discernible labeled structures. Finally, correlation of the pixel intensity of both proteins was determined using Coloc 2 plugin of Fiji software, with Pearson’s Correlation Coefficient (PCC) used to quantify the co-localization. Except where indicated, eight to ten cells were evaluated for each condition (2–4 independent experiments).

### *In vivo* time-lapse microscopy

For *in vivo* time-lapse microspcopy studies, fluorescence images, corresponding to mCherry-Rab1b and YFP-Sec24 expression, were both taken at 4 second intervals for 10 min at 0.05% laser power (acquisition time between channels was between 0.5 to 1.5 s, according to the fluorescence intensity of the sample). Fluorescence images corresponding to dual expression of mCherry-Rab1b and GFP-p115 were taken at 6 second intervals for 6 min at 0.05% laser power. Images from triple transfection (mCherry-Rab1b, YFP-Sec24 and SialT2-CFP), were obtained at 30 second intervals for 30 min at 0.05% laser power. One optical Z slice was visualized and imaged using a PLAPON 60XO (1.42 NA) on an Olympus IX81 microscope equipped with a spinning-disk confocal unit (slit mode) DSU (Olympus) and an Orca ER camera (Hamamatsu) under cell^**R**^ software control (Olympus Imaging, Center Valley, PA, USA.). During imaging, cells were maintained at 37°C in an incubation chamber (Harvard Instruments).

### Quantitative analysis of variance of pixel fluorescence intensity (PFIVar)

Time-lapse images corresponding to triple transfection were taken for at least 10 min, and an area including stable and single COPII structures was selected employing the ROI (region of interest) manager of Fiji software. The mean fluorescence intensity of pixel (PFI) and the corresponding standard deviation (SD) of Rab1b and cargo were measured in this area at different times. PFI variance values were then calculated as previously described [28], and normalized by dividing all variance values by the initial one (at *t* = 0). Finally, relative PFI variance was calculated by dividing by the relative mean of the fluorescence intensity of the corresponding COPII structure, which was used as a reference focal plane.

### FRAP analysis

Fluorescence recovery after photobleaching (FRAP) assays were performed in cells co-expressing mCherry-Rab1b (wild type) and YFP-Sec24 or expressing mCherry-Rab1b (wild type), YFP-Sec24 and GBF1-E794K. The GBF1-E794K-myc transfected cells were detected because they exhibited a distinctive and complete mCherry-Rab1b labeled punctate pattern (see text for more explanation), with punctate mCherry-Rab1b labeled structures co-localizing with YFP-Sec24 being selected to perform FRAP. For mCherry-Rab1b FRAP analysis, two prebleach images were taken at a 0.05% laser intensity. Then, the region of interest (ROI) was bleached for 15 s at 100% laser intensity, with fluorescence recovery in the bleached area being monitored by scanning at 10% laser intensity in a free run mode (no interval) using a FV1000 Olympus confocal scanning microscope. At the same time periods, the intensity of a reference region (RER) was recorded, with the RER being an area selected outside of the ROI. Average fluorescence intensities were measured using the Fiji software (http://fiji.sc/Fiji, NIH). At each time point, the percentage of fluorescence recovered in the ROI was plotted using the following equation: F(t) = (F_ROI_/F_RER_)/(F_preROI_/F_preRER_)×100, where F_preRER_ is the fluorescence intensity of the RER before bleaching, F_RER_ is the fluorescence intensity in the RER at time t, F_preROI_ represents the ROI intensity before bleaching, and F_ROI_ is the ROI intensity at time t. FRAP assays were performed in cells expressing similar levels of Cherry-constructs. For each quantitation, the number of cells imaged is indicated by “n”, which refers to individual cells from at least three independent transfections. Fluorescence intensity within the entire bleached region was quantified using Fiji software (http://fiji.sc/Fiji). A circular area approximately double to triple the size of the particle of interest was selected such that the chosen structure did not move outside this area during the experiment. Finally, FRAP images were presented graphically using Microsoft Excel (Microsoft). During imaging, dishes were maintained at 35 ± 2°C (Tritech DigiTherm temperature controller) in a stage top incubator (INU, Tokai Hit).

### BFA and Nocodazole treatments

For immunofluorescence assays, HeLa cells were incubated with a medium containing 5 μg/mL BFA (Sigma-Aldrich, St. Louis, MO, USA) for 30 min at 37°C. BFA washout (BFA WO) was carried out by removing, rinsing, and incubating cells with fresh medium at 37°C. Immunofluorescence assays were carried out at each time point by labeling cells with antibodies. For *in vivo* studies, cells were treated with 1 μg/mL of BFA for 2 h at 37°C. In time-lapse assays with triple transfection, images were taken after 10 min of BFA washout.

To depolymerize microtubules, cells were chilled on ice for 30 min before being warmed in complete medium containing 2.5 μg/mL nocodazole.

### Statistical Analysis

For quantitative co-localization analysis, parametric data are shown as the mean value of PCC ± standard deviation (SD). The statistical significance between means of different conditions was measured by the Student’s t-test using Graphpad Prism 5.0 version (http://www.graphpad.com).

For co-localization time analysis, non-parametric data are shown as the median value of the co-localization time with interquartile ranges (Percentiles 25% and 75%). The statistical significance between medians of different conditions was measured by a one-way ANOVA followed by Dunn’s Multiple Comparison method (Graphpad software). Differences with P < 0.05 were considered as being statistically significant.

## Results

### Quantitative co-localization analysis between Rab1b and different markers at steady state and after BFA treatment

Rab1b participates in ER to Golgi transport and co-localizes with ERES, VTC structures and the Golgi complex. In addition, Rab1b interacts with some proteins located on these structures such as Sec23, p115, and GM130, respectively. Rab1b also participates, through its interaction with GBF1 (the exchange factor of Arf1 at the ER-Golgi interface), in retrograde Golgi to ER transport mediated by COPI vesicles. However, quantitative information about co-localizations between Rab1b with ERES or VTCs markers and how they might change under different transport conditions remains unknown. We assume that the proportion of Rab1b associated to ERES or VTCs at steady state (a situation that implies a balance between anterograde and retrograde membrane transport) is modified when the transport balance is perturbed. To test this hypothesis, we performed quantitative co-localization studies of Rab1b with different markers, in the presence or absence of Brefeldin A (BFA). BFA blocks anterograde ER-Golgi transport and induces Golgi redistribution to the ER, and therefore all the cargo proteins accumulate there [29, 30]. We selected BFA to alter transport between the ER and the Golgi over other treatments (incubation at 15°C for example), because BFA blocks COPI membrane association without affecting COPII recruitment. Thus, Rab1b activity is stopped after its association with COPII structures before Arf1 activation, a step required for COPI recruitment.

**Fig 1:**
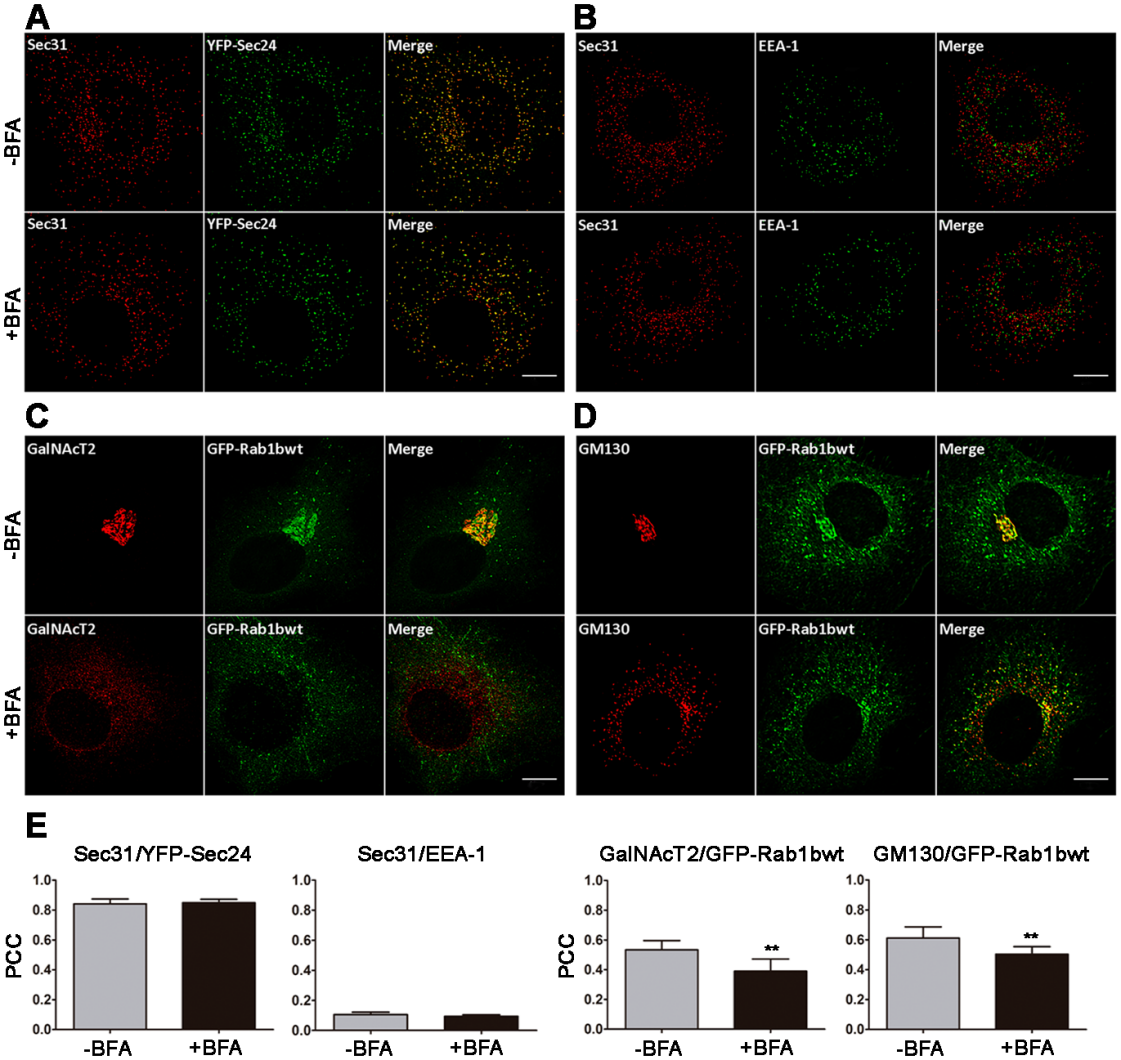
Quantitative co-localization analysis of different intracellular transport markers in control (-BFA) and BFA-treated cells (+BFA). A-D) Confocal microscopy images of HeLa cells. Anti-GFP antibody and antibody against the indicated endogenous marker were used. A) Positive control with COPII markers Sec31 and YFP-Sec24wt. B) Negative control using an early endosome marker (EEA-1) and the COPII marker Sec31. C and D) Co-localization of the Golgi markers GalNAcT2 or GM130 with GFP-Rab1b, respectively. Scale bars represent 10 μm. E) Bar graphs depicting the Pearson correlation coefficient (PCC) of co-localization between the different markers (8 to 10 cells per condition, from 2–3 independent experiments). Data are expressed as mean ± SD (_**_ = p<0.001).

We performed co-localization analysis using Pearson´s correlation coefficient (PCC, see Material and Methods), and cells expressing low to medium levels of the GFP variants were selected for all analysis. For the positive control, the co-localization of two COPII markers (Sec24 and Sec31) was analyzed in HeLa cells transiently transfected with YFP-Sec24 (Fig 1A), and anti-Sec31 and anti-GFP antibodies were used to quantify the co-localization between them. A negative control was performed by analyzing the co-localization between Sec31 and the endosome marker EEA-1 (Fig 1B). The PCC between Sec24 and Sec31 (0.84±0.03) and between Sec24 and EEA-1 (0.11 ±0.02) demonstrated high and low degrees of co-localization, respectively. Moreover, neither PCC value were significantly modified after BFA treatment (Fig 1E). Cells were incubated with BFA, 5 μg/mL, for 2h to ensure complete BFA response of the entire cell population. Next, we analyzed co-localization between GFP-Rab1b with the medial/trans Golgi marker GalNAcT2 and the cis-Golgi marker GM130. As previously reported, GFP-Rab1b was localized at the juxtanuclear region and at the peripheral punctate structures (Fig 1C and 1D, [23], while GalNacT2 and GM130 were concentrated mostly at the juxtanuclear region. The PCC between Rab1b and GalNacT2 and between Rab1b and GM130 corroborated these incomplete overlaps (0.53±0.06 and 0.61±0.07 respectively, Fig 1E). These PCCs were lower than those showing co-localization between the two COPII markers, but higher than the one observed for the negative control Sec31 and EEA-1.

After BFA treatment, GalNacT2 displayed a diffuse reticular pattern, thus indicating its redistribution to the ER in a similar way to that exhibited by the transmembrane Golgi proteins (ie. ManII). Unlike GalNacT2, after BFA treatment, Rab1b and GM130 relocated to punctate peripheral structures (Fig 1D). In agreement with this differential phenotype, co-localization between Rab1b and GalNAcT2 decreased after BFA treatment (from 0.53±0.06, to 0.39±0.08). The Rab1b punctate pattern after BFA treatment showed a range of responses. For instance, the GFP-Rab1b signal completely relocated from the juxtanuclear Golgi pattern to the peripheral punctate structures after BFA treatment. Furthermore, some cells exhibited peripheral punctate structures plus a diffuse (ER-like) pattern, or alternatively, it was possible to detect peripheral punctate structures plus a slight signal at the juxtanuclear region likely to represent the accumulation of some of the punctate structures in the region. However, we cannot exclude the possibility that the juxtanuclear signal might represent an incomplete Golgi disassembly in response to BFA. Interestingly, despite both GM130 and Rab1b being relocated to punctate structures, their degree of co-localization slightly decreased after BFA treatment (from 0.61±0.07 to 0.50±0.05) indicating that they partially redistributed to different structures subsequent to BFA treatment.

**Fig 2:**
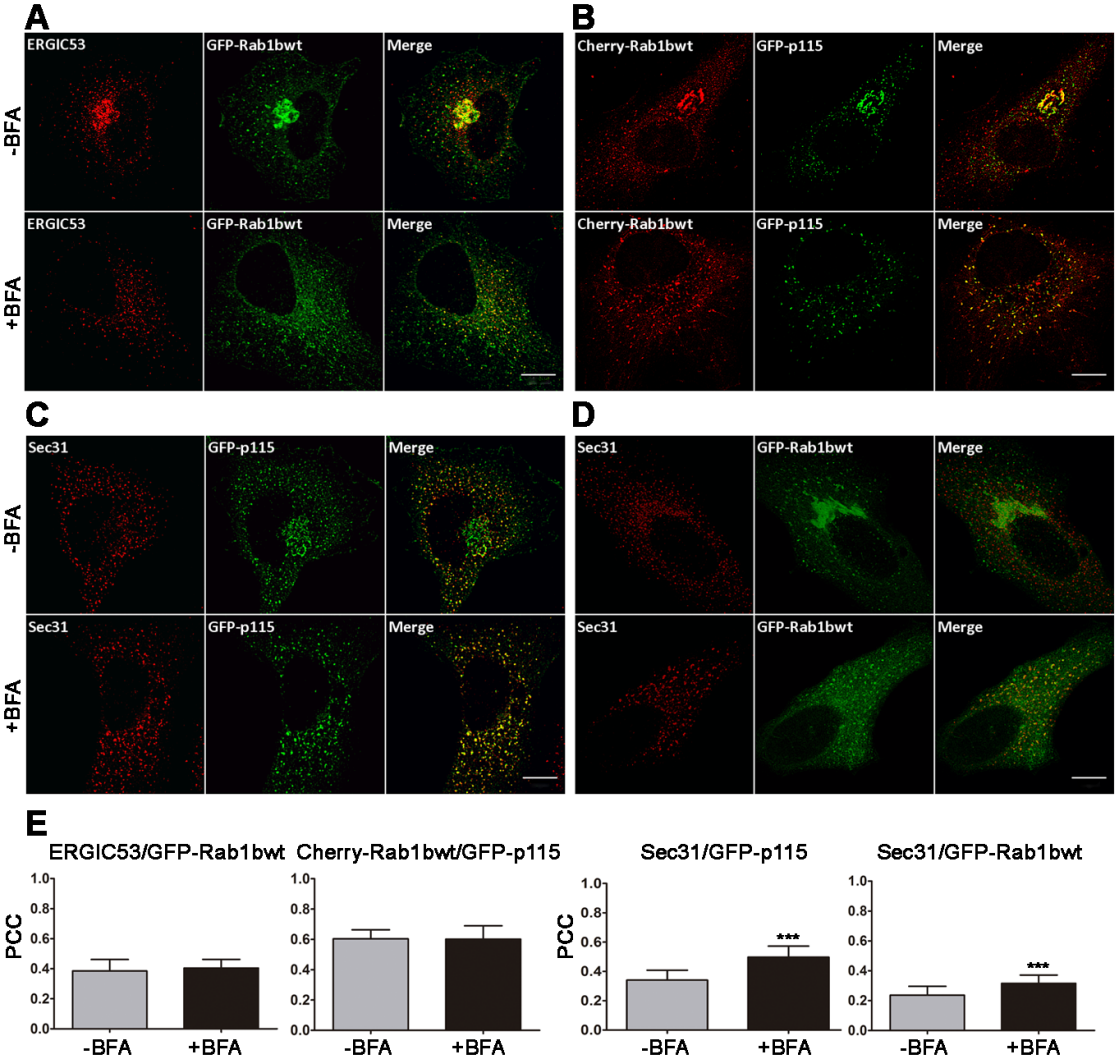
Quantitative co-localization analysis of different intracellular transport markers in control (-BFA) and BFA-treated cells (5 ug/mL for 2h). (A-D) Confocal microscopy images of transfected HeLa cells (A, C and D). Immunofluorescence assays were performed with anti-GFP antibody (to detect GFP-Rab1bwt or GFP-p115) and an antibody against the indicated endogenous marker (ERGIC53 or Sec31). (B) Direct fluorescence detection of the mCherry or GFP indicated fusion proteins. Scale bars represent 10 μm. (E) Bar graphs depicting the Pearson correlation coefficient (PCC) of co-localization between the different markers (8 to 10 cells per condition, from 2–3 independent experiments). Data are expressed as mean ± SD (_***_ = p<0.0001).

At steady-state Rab1b was distributed at ERES, VTCs and the Golgi complex. BFA induces Golgi redistribution to the ER, then an amount of Rab1b associated to Golgi should relocate to VTCs and/or ERES in response to BFA. To test this assumption, we quantitatively analyzed Rab1b co-localization with ERES and VTCs markers in both, control and BFA-treated cells (Fig 2). First, we analyzed Rab1b redistribution to VTCs using ERGIC53 [1] as a marker. As shown in Fig 2A, ERGIC53 and Rab1b had similar immunofluorescence patterns, displaying a high concentration in the juxtanuclear region and punctate structures. In response to BFA, both proteins were fully distributed to punctate structures, with the Rab1b-labeled structures being more dispersed to the cell periphery than ERGIC53-labeled structures, and also exhibiting a slight reticular pattern. Co-localization between Rab1b and ERGIC53 (PCC = 0.39 ± 0.08) was not significantly modified by the BFA treatment (Fig 2E). Moreover, Rab1b co-localization with p115, a Rab1b effector localized at the ER-Golgi interface was analogous in both conditions (-BFA and +BFA). Also, Rab1b/p115 co-localization (PCC = 0.60 ± 0.06) was higher than Rab1b/ERGIC53 (PCC = 0.39 ± 0.08). These results were consistent with their cellular distribution, as ERGIC53 is considered a bona fide VTCs marker, while p115, as well as Rab1b, were localized at the VTCs and cis-Golgi. Next, we analyzed both p115 and Rab1b redistribution to COPII after BFA treatment. In both cases, the co-localization with the COPII structures increased. For p115/Sec31, the PCC changed from 0.34 ± 0.07 to 0.50 ± 0.08 after BFA, while for Rab1b and Sec31 co-localization, the PCCs showed a slight but significant increase (from 0.24 ± 0.06 to 0.32 ± 0.06). It has been reported that, after BFA treatment, ERGIC structures get closer to ERES [31], so we cannot exclude the possibility that the increased co-localization of p115 with Sec31, after BFA treatment, is because the short distance separating the structures labelled with these markers is poorly distinguished by our approach. However, despite this drawback, the co-localization of Rab1b with Sec31 is still low after BFA treatment (PCC~ 0.30), and even lower than the co-localization of P115 with Sec31 (PCC~0.50) in the same condition. The fusion with GFP or mCherry could modify Rab1b localization, however we consider this option unlikely since endogenous Rab1b showed similar immunofluorescence patterns to the GFP-Rab1b variant (S1 Fig), and quantitative co-localization between endogenous Rab1b and GM130 (our unpublished result) exhibited similar PCC values to the fluorescent variants in both the control and BFA treated cells. Taken together, the data suggest that a fraction of Rab1b co-localized with COPII structures, even when all cargo proteins were accumulated at the ER.

### Dual color visualization of Rab1b with COPII (Sec23) or p115 in living cells

Previous time-lapse microscopy studies performed with GFP-Sec23, GFP-p58 or GFP-p115 showed the dynamic behavior of the COPII, VTCs and p115-labeled structures, respectively. COPII structures are relatively stable and generally immobile [3, 32], whereas VTCs and p115-labeled structures are highly mobile, segregate from COPII/ERES and move towards the Golgi. In addition, punctate GFP-Rab1b labeled structures exhibited a combined behavior between COPII and VTCs structures [33]. Although individual dynamics studies of COPII, VTCs, p115 or Rab1b have been previously reported, information on dual time-lapse studies in living cells is rather limited.

**Fig 3:**
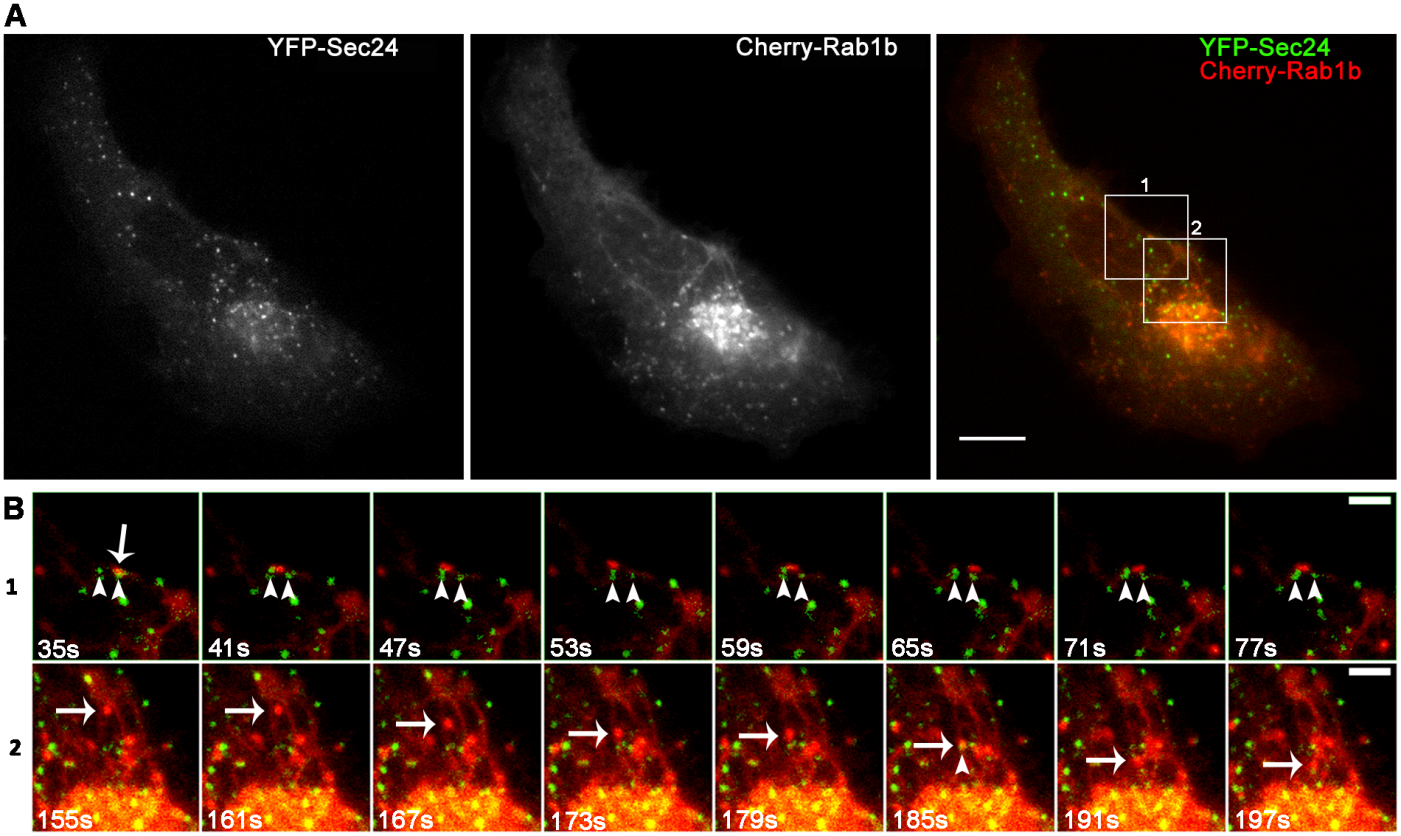
Representative time-lapse imaging of HeLa cells co-transfected with mCherry-Rab1bwt and YFP-Sec24wt (S1 Movie). (A) Confocal images showing individual Sec24 and Rab1b signals and their overlap (B 1–2) Magnification of different ROIs shown in A at the indicated time points: 1) Rab1b-labeled structures (arrow) moving between two Sec24-labeled stable structures (arrowheads). 2) Rab1b—labeled punctate structures moving towards the Golgi complex (arrow) and passing through a stable Sec24-labeled structure (arrowhead 185 sec). Data represent at least three independent experiments. Scale bars represent 10 μm (A) and 3 μm (B).

**Fig 4:**
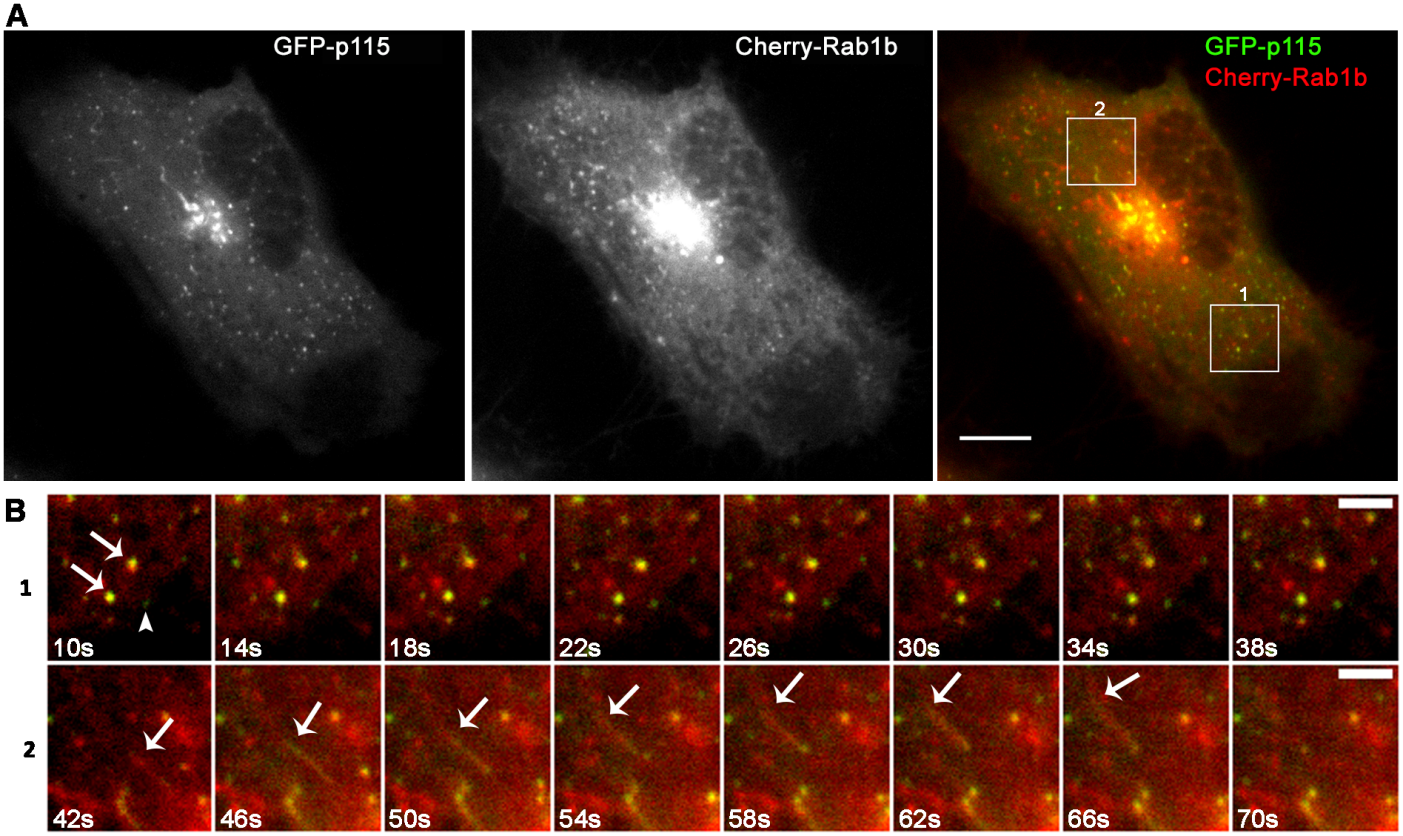
Representative time-lapse imaging of HeLa cells co-transfected with mCherry-Rab1bwt and GFP-p115wt (S2 Movie). (A) Confocal images showing individual p115 and Rab1b signals and their overlap (B 1–2) Magnification of different ROIs shown in A at the indicated time points: 1) Punctate structures labeled with both Rab1b and p115 (B1, arrows) which remained stable throughout the recorded time (more than 6 minutes, 392sec). It was also possible to detect p115-labeled structures that did not co-localize with Rab1b over the same period of time (B1, arrowhead); 2) tubular structure labeled with both p115 and Rab1b originated from the Golgi and rapidly moving towards the cell periphery until it vanished (B3, arrow). Data represent at least three independent experiments. Scale bars represent 10 μm (A) and 3 μm (B).

Here, we analyzed simultaneously the dynamics of Rab1b with COPII or Rab1b with p115-labeled structures with the aim of improving the understanding of the spatial-temporal dynamics of Rab1 overlap with COPII or p115-labeled structures. *In vivo* time-lapse microscopy assays of HeLa cells co-transfected with Cherry-Rab1b and YFP-Sec24 (Fig 3, S1 Movie) or with mCherry-Rab1b and GFP-p115 (Fig 4, S2 Movie), were carried out for 6 to 10 minutes using confocal microscopy with a spinning disk, and images were taken every 4 to 6 sec. To understand better the role of Rab1b at ERES/VTCs, we focused our dynamics analysis on peripheral punctate structures.

As previously reported [33], Rab1b-labeled punctate structures were relatively mobile with limited-range movements and exhibited few tubular patterns. In contrast, COPII punctate structures were mostly immobile and stable. Live-cell dual color imaging indicated that Sec24 and Rab1b co-localization was mostly transient, with some examples of their overlaps being displayed in Fig 3 and S1 Movie.

After analysis of the time-lapse assays, the key points to emphasize are that, in agreement with their co-localization degree (Fig 2, PCC: 0.24 ± 0.06), most of the YFP-Sec24 labeled structures did not associate with mCherry-Rab1b, and only a low fraction of YFP-Sec24 structures overlapped transiently with Rab1b. This transient overlap was mostly due to the high mobility of the Rab1b-labeled structures.

We also performed similar dual expression time-lapse assays in cells transfected with mCherry-Rab1b and GFP-p115. The p115-labeled punctate structures were found to be highly mobile and described trajectories from and towards the Golgi complex. It was also possible to observe long-lived stable p115-labeled punctate structures. Moreover, p115-tubular like structures exhibiting both long-lived or mobile behaviors were often detected (Fig 4, S2 Movie). The observation of the multiple movies revealed that most of the Rab1b tubular-like structures co-localized with similar p115 labeled structures. However, there were p115-tubular structures without any Rab1b label on them.

Taken together, these dual expression time-lapse assays confirm that the Rab1b/Sec24 and Rab1b/p115 overlaps were mostly transient and suggest that they might occur at the same time in spatially distinct steps of ER to Golgi transport.

### Spatial-temporal analysis of Rab1b overlap with ERES during anterograde transport of cargo

The BFA effect is reversible, with its removal by changing the incubation media (washout) allowing the recovery of anterograde transport and triggering reorganization of the Golgi apparatus. During the early stages of BFA washout (30–45 min), the anterograde cargo transport is synchronized and Golgi proteins concentrate in ERES in a Rab1b dependent manner [24]. In order to improve the understanding of the spatial and temporal overlap of Rab1b with COPII structures during anterograde transport of cargo, we performed time-lapse assays during the early stages of BFA washout (~30–40 min) in HeLa cells co-transfected with mCherry-Rab1b, YFP-Sec24 and SialT2-CFP. The SialT2 glycosyltransferase is required for the synthesis of gangliosides and is localized at the proximal Golgi, and relocates to the ER after BFA treatment [34]. We used SialT2-CFP as a marker of a cargo protein in order to follow ER to Golgi transport during early BFA washout times. The SialT2-CFP construct includes the N-terminal domains of SialT2 (amino acids 1–57) necessary for Golgi localization [35].

**Fig 5:**
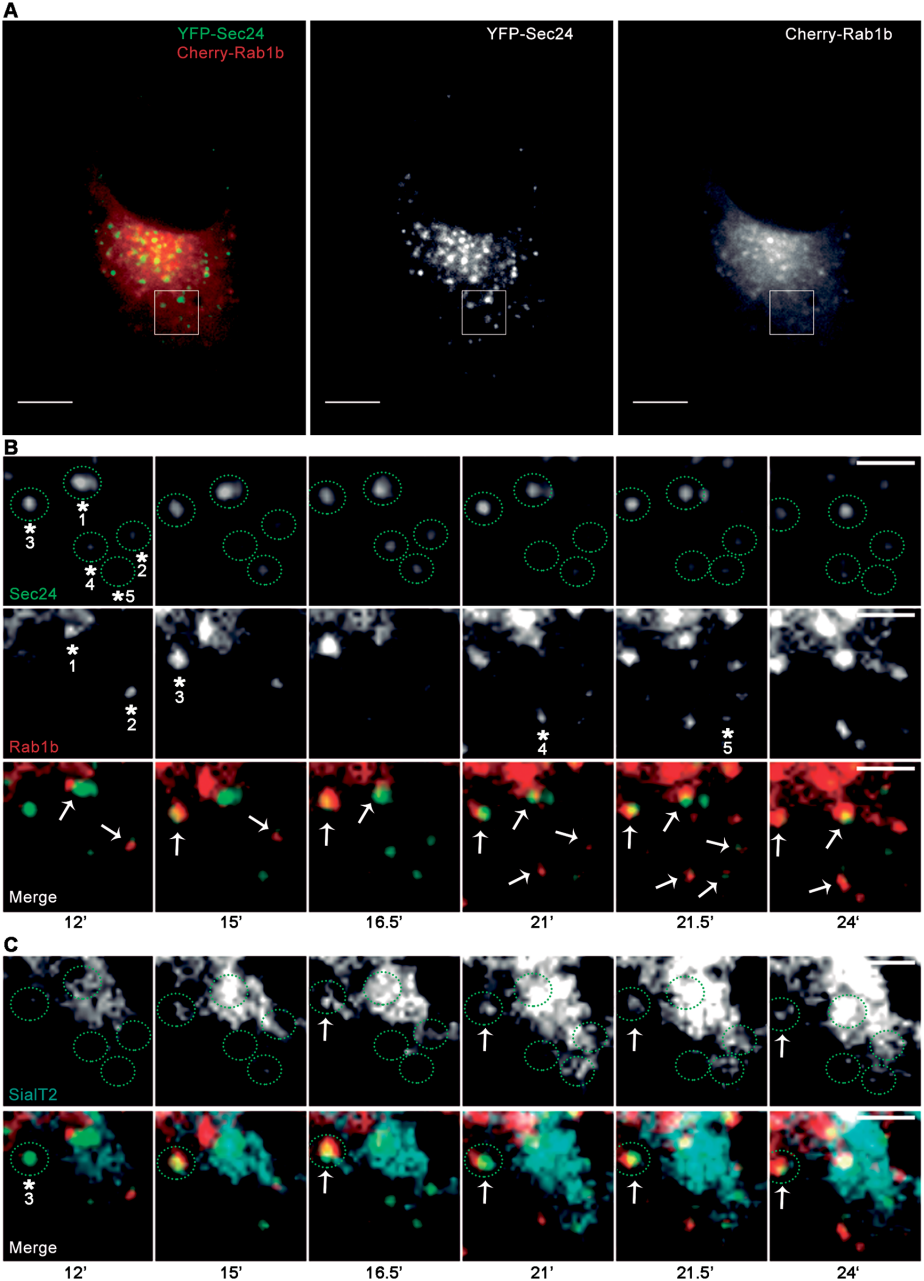
Representative time-lapse imaging of HeLa cells co-transfected with mCherry-Rab1b, YFP-Sec24 and SialT2-CFP during BFA washout. After 24h of transfection, cells were incubated with BFA (1 μg/mL for 2h), then BFA was removed (washout) and *in vivo* time-lapse assays were performed. (A) Confocal image showing the overlap of YFP-Sec24 and mCherry-Rab1b and their individual signals. (B) Magnification of ROIs indicated in A, showing panels of the individual YFP-Sec24 and mCherry-Rab1b signals and their merge at the indicated times. Asterisks in Sec24 panel indicate five structures (labeled from 1 to 5, at the first indicated time, and with green circles at all time points) that remained stable at the indicated times. Asterisk in Rab1b panel indicates the first time that Rab1b associated to one of the Sec24 labeled structures. Arrows in the merge panel indicate structures where Sec24 and Rab1b are associated at the indicated times. (C) Magnification of same ROIs and times indicated in A and B respectively. Panels show individual SialT2-CFP signal and the merge including YFP-Sec24, mCherry-Rab1b and SialT2-CFP, and green circles specify the location of the same YFP-Sec24 structures indicated in B. Arrows in SialT2-CFP panel indicate the SialT2 concentration/sorting. Asterisk and circle in the merge panel indicate the Sec24 stable structures (#3), where the SialT2-CFP signal is concentrated at the indicated times (arrows). These arrows also show YFP-Sec24, mCherry-Rab1b and SialT2-CFP co-localization. Scale bars represent 10 μm (A) and 3 μm (B and C).

Time-lapse assays were performed approximately 10 minutes after BFA washout, capturing YFP, mCherry, and CFP images for 30 second intervals during 30 minutes. Images were then analyzed, and YFP-Sec24 labeled structures (50 structures, n = 4 cells from independent experiments) that remained stable for 7 to 15 minutes were selected to describe the mCherry-Rab1b association to these structures. A representative analysis is shown in Fig 5, where five YFP-Sec24-labeled structures that remained stable from the 12^th^ minute onwards (from minute 12 to 24 after washout) are indicated (asterisks 1–5 and green circles, Sec24 panel). As shown in the Rab1b panel, during these 12 minutes, mCherry-Rab1b associated to all structures but at different times. The first moment of Rab1b association to each Sec24 structure is indicated with an asterisk, with the same asterisk number associated to its respective Sec24 structure. In some Sec24 structures, Rab1 associated recurrently during the time of the movie.

We also studied Rab1b and COPII overlapping relative to cargo exit by analyzing SialT2-CFP behavior in the same COPII structures described above (Fig 5C, SialT2-CFP panel). At the start of BFA washout, SialT2 was located at the ER and displayed a reticular pattern, which later started to concentrate at the ERES (labeled with Sec24, Fig 5C, arrows in SialT2-CFP and merge panels). SialT2-CFP concentration also co-localized with Rab1b as shown for the Sec24 structure labeled as #3 in panel B at times 16.5 and 24 min (Fig 5C, arrows, merge panel). In this case, the SialT2-CFP concentration was detected in 1 of the 5 COPII structures. It is important to mention that the SialT2-CFP diffuse but intensive pattern enclosed in the circular area corresponding to the Sec24 structure #1 was not considered a SialT2-CFP concentration according to the quantification explained below, with this pattern corresponding to SialT2-CFP distributed to the ER.

**Fig 6:**
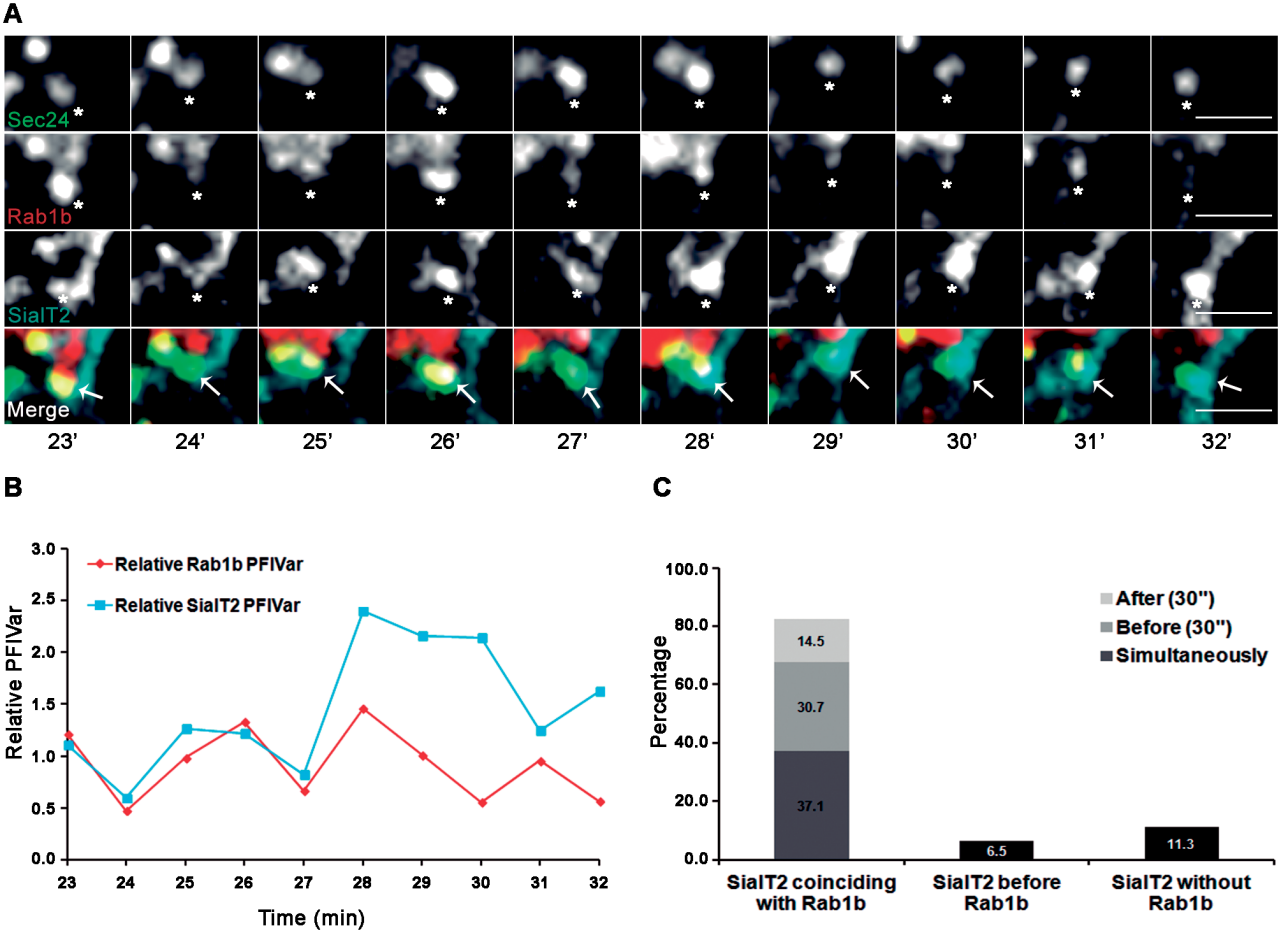
Representative time-lapse imaging of HeLa cells co-transfected with mCherry-Rab1b, YFP-Sec24 and SialT2-CFP during BFA washout. After 24h of transfection, cells were incubated with BFA (1 μg/mL for 2 h), then the BFA was removed (washout) and the time-lapse assays were performed. (A) Confocal image showing the individual YFP-Sec24, mCherry-Rab1b and SialT2-CFP signals and their merge at the indicated time points. Scale bars represent 3 μm. (B) Time-dependent changes in relative PFIVar of Rab1b or SialT2, at the region of interest depicted in A, calculated as described in Materials and Methods. (C) Bar graph depicting the percentage of the SialT2-CFP PFIVar peak behaviors (n = 62 events) clustered into three different groups: The first group containing SialT2 peaks coinciding with high Rab1b levels (named SialT2 coinciding with Rab1b), with this group including the SialT2 PFIVar peaks occurring simultaneously, 30 seconds before or after the Rab1b PFIVar peaks. In the second group were included the SialT2 PFIVar peaks which occurred completely before a Rab1b peak (named SialT2 before Rab1b), and finally, the third group collected all the SialT2 PFIVar peaks in the absence of Rab1 (named SialT2 without Rab1b).

To analyze accurately the spatial-temporal correlation of SialT2 sorting relative to the Rab1b concentration at ERES structures, we quantified the SialT2 and Rab1b concentrations at different time points for each YFP-Sec24 structure previously mentioned (corresponding to the 50 structures that remained stable from 7 to 15 minutes). The approach used to quantify sorting/concentration was similar to that described to quantify VSV-G sorting at ERES, by which changes in fluorescence-intensity variance were measured in living cells [28]. Specifically, we analyzed the time-dependent change in the variance of the pixel fluorescence intensity (PFIVar) of mCherry-Rab1b or SialT2-CFP in an area including an identified ERES structure (labeled with YFP-Sec24) during the initial BFA washout period. All PFIVar values were expressed relative to both the PFIVar at the initial time and to the relative mean of fluorescence intensity of the corresponding COPII structure (to avoid changes due to variations on the focal plane, Material and Methods). Fig 6 shows a representative quantification of Rab1b and SialT2 PFIVar in an YFP-Sec24-labeled structure localized in the cell periphery (Fig 6A), where typically, during the course of the experiment, the PFIVar for both proteins increased until reaching a maximum value before decreasing (Fig 6B). The SialT2 PFIVar peaks revealed dissimilar values, which were sometimes shifted in time relative to the Rab1b PFIVar peak. All the 50 YFP-Sec24 labeled structures that we analyzed (n = 4 cells from independent experiments) showed a Rab1b PFIVar peak at some point of the analysis. However, only 30 of these had at least one SialT2 PFIVar peak, but revealed 62 SialT2-CFP concentration events. Analyses of the time-dependent changes in PFIVar (Fig 6C) for both proteins (mCherry-Rab1b and SialT2-CFP) for the 62 events indicated that in some of these (~37%) the SialT2 and Rab1b PFIVar peaks occurred at the same time, while in ~14 and 30% of the events, the SialT2 PFIVar peaks happened 30 seconds before or after Rab1b PFIVar, respectively. It is important to remark that in these two circumstances, although the peaks in the PFIVar curves were shifted in time, there was a significant overlap in the overall curve. Therefore, in all these cases (~82%), the SialT2 concentration events corresponded to a high Rab1b relative PFIVar. A small number of events occurred before Rab1b concentration (~6%) or without any Rab1b intensity modification (~11%).

In conclusion, the SialT2 concentration on COPII structures (defined as change in the variance of pixel fluorescence intensity) was mostly detected in COPII/Rab1b positive structures, but not all COPII/Rab1b structures concentrated SialT2 during the analyzed times. Moreover, SialT2 concentration at ERES was a transient event, and it was typical to observe SialT2 concentration points appearing and vanishing at random times at ERES structures.

### Analysis of Rab1b overlap with COPII-labeled structures in cells with impaired GBF1 activity

We next aimed to analyze Rab1b dynamics behavior in punctate structures labeled with both Rab1b and YFP-Sec24 after blocking COPI recruitment using BFA to inhibit the activity of GBF1, a Rab1 effector required for COPI recruitment. GBF1 and ARF-GDP interact to form a complex that is stabilized on ER membranes in BFA-treated cells [36]. Hence, BFA inhibits GBF1 exchange activity on Arf1 [37], blocks COPI recruitment and induces redistribution of Golgi to the ER. Although BFA blocks COPI recruitment, COPII and Rab1b are still associated to ERES after BFA treatment (Fig 2A). At the ERES-Golgi interface, Rab1b regulates COPII dynamics [24] and recruits GBF1, thus the Rab1b-labeled structures induced after BFA treatment represent the first site of Rab1b action during ER to Golgi transport. GBF1 is a Rab1b effector, but it is unknown if the dynamic of Rab1b is regulated by GBF1 activity.

**Fig 7:**
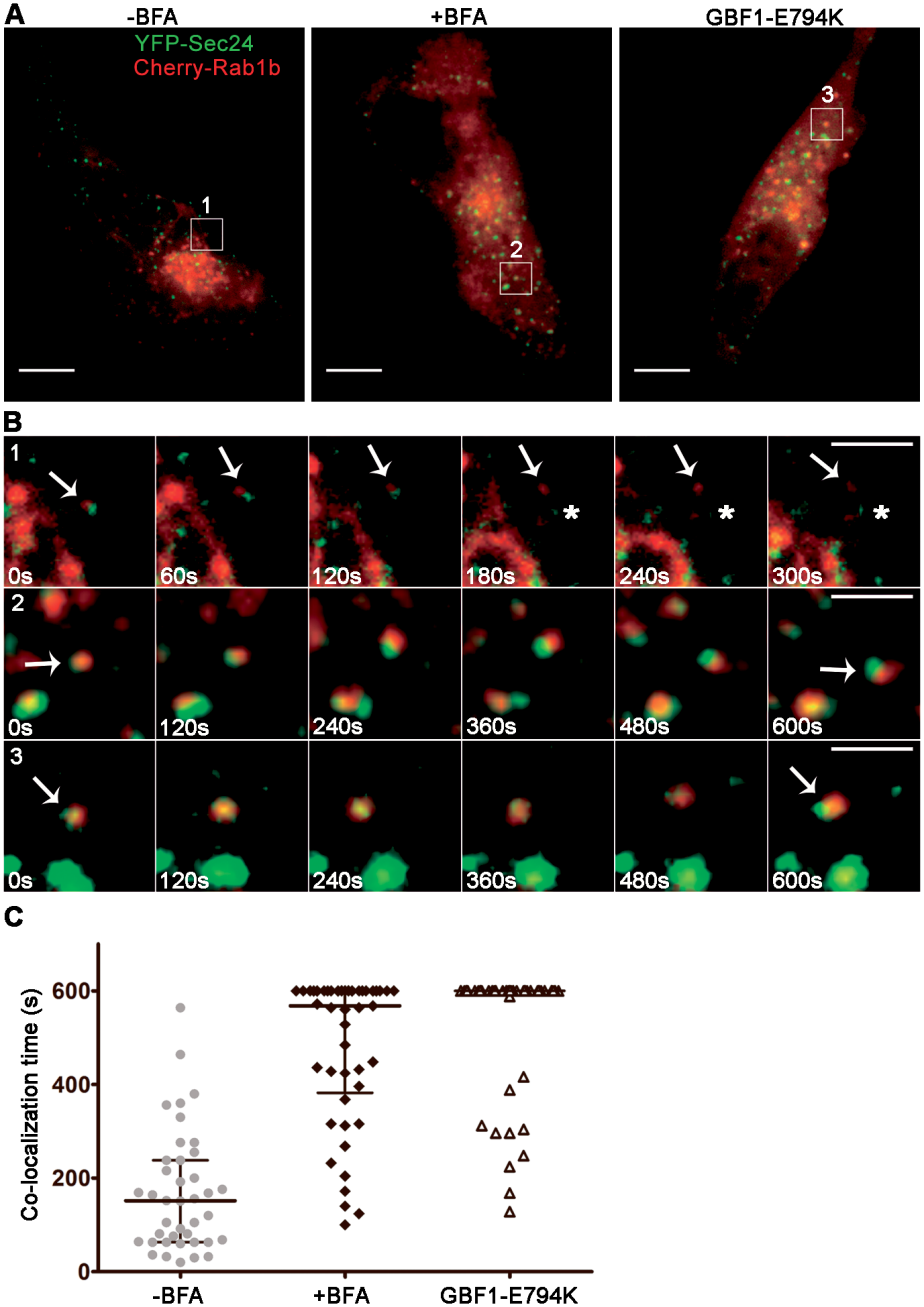
Co-localization time of Rab1b with ERES structures. Dual color time-lapse assays were performed in cells expressing YFP-Sec24 and mCherry-Rab1bwt (S3 Movie). (A) Representative images of the time-lapse assays performed in HeLa cells for three different conditions: Control (-BFA), BFA treated cells (+BFA, 5 μg/mL for 2h), and in cells also co-transfected with GBF1-E794K-myc. (B, panels 1–3) Magnification of the different ROIs shown in A at the indicated time points of the time-lapse assay. Arrows indicate the Rab1b- labeled structure analyzed in the example. Asterisks in 1 indicate the times that the Rab1b-labeled structure is no longer associated to the Sec24-labeled structure. Scale bars represent 10 μm (A) and 3 μm (B). (C) Scatter plot of the co-localization time for cells at the indicated conditions. Sec24 and Rab1b-labeled structures were randomly selected for the analysis for each condition (-BFA: 40, +BFA: 45, GBF-E794K: 51 structures, n = 3 cells per condition).

Double-label time-lapse assays were performed to analyze the permanence time of Rab1b on ERES structures. Time-lapse studies of HeLa cells co-transfected with mCherry-Rab1b and YFP-Sec24 were performed for 6 to 10 minutes using confocal microscopy with spinning disk and images taken every 4 to 6 seconds. The overlap time between Rab1b and ERES (co-localization between mCherry-Rab1b and YFP-Sec24) was measured in both control and in BFA-treated cells. We randomly selected 50 structures with double label at the first frame (time 0 sec) and analyzed co-localization of mCherry-Rab1b with YFP-Sec24 over the total time. It was taken into account that even though COPII subunits undergo multiple rounds of binding and dissociation to membranes, they are relatively immobile structures, whereas Rab1b labeled structures are highly mobile. Therefore, we measured the intensity of the Rab1b punctate structures relative to that of their respective COPII co-localizing structures over the period of the time-lapse assay. Since we analyzed Rab1b structures co-localizing with COPII in the first frame, and also due to the high mobility and transient behavior of Rab1b structures, it was necessary to establish a criterion to measure co-localization. Thus, we considered that when the relative Rab1b/Sec24 intensity value was lower than 40% of the initial relative intensity value (time 0), then the Rab1b intensity had dropped sufficiently to be able to assume that Rab1b and Sec24 were no longer co-localizing, especially in the case of the Rab1b structures that had vanished. For Rab1b mobile structures, the Rab1b/Sec24 relative intensity value was even lower than 40% of the initial value. It is important to mention that we focused the analysis on the time of the Rab1b/Sec24 co-localization (including partial co-localization), and not on the degree of overlapping between the Rab1b and Sec24 signals under different conditions. As shown in Fig 7, the Rab1b/Sec24 co-localization time increased approximately 4 times in BFA treated cells compared to the control cells (with a median increase of ~150 to 570s). It was necessary to increase the brightness in the merge images (Fig 7, panel A) to clearly exhibit the punctate structures presented below in Fig 7B. Examples of the representative levels of expression used for in vivo double-label time-lapse assays are observed in the single channels exhibited in the supporting, S1 and S2 movies.

GBF1 is not the only BFA target, as BFA can also induce ADP-ribosylation of BARS (brefeldin A-ADP-ribosylated substrate), a bifunctional protein with a role in the nucleus as a transcription factor and in the cytosol as a regulator of membrane fission during intracellular trafficking [38, 39]. To investigate if changes in the co-localization time were a specific effect of BFA on GBF1, a dominant negative GBF mutant, E794K, was expressed in HeLa cells. This mutant lacks the ability to exchange GDP for GTP on ARF1, since it carries a mutation in the catalytic glutamate residue and is defective in nucleotide exchange activity. GBF1-E794K expression induces a BFA-like phenotype with COPI cytosolic redistribution [8], and causes a complete disassembly of the Golgi. Then, Golgi proteins redistribute to the ER or to the peripheral punctate structures that contain GBF1-E794K (like p115 and GM130). Rab1b is also redistributed to punctate structures in GBF1-E794K expressing cells (Fig 7).

Triple transfections were then performed to analyze the effect of GBF1-E794K on the Rab1b/COPII co-localization time. HeLa cells were co-transfected with GBF1-E794K-myc, mCherry-Rab1b and YFP-Sec24. First, immunofluorescence assays were performed to confirm the triple transfection efficiency, by using suitable lasers and filter combinations to detect an anti-myc mouse antibody labeled with an Alexa anti-mouse (specifically to label GBF1-E794K-myc) together with mCherry-Rab1b and YFP-Sec24. We established the conditions (DNA ratio of the constructs) to obtain more than 80% triple transfection, and confirmed that all GBF1-E794K-myc transfected cells exhibited a distinctive and complete mCherry-Rab1b labeled punctate pattern (in the absence of a juxtanuclear Golgi mark). In contrast, untransfected cells had a normal juxtanuclear Golgi concentrated pattern mixed with a punctate pattern (data not shown). We performed time-lapse microscopy studies in cells displaying a complete punctate mCherry-Rab1b pattern in order to identify E794K-myc transfected cells. Imaging acquisition and co-localization time analysis were carried out as described above for double transfected cells. As shown in Fig 7A–7C, the Rab1b/Sec24 co-localization time in E794K expressing cells was at least four time longer than in control cells, and many Rab1b/COPII labeled structures remained co-localized at the end of the time-lapse assay.

**Fig 8:**
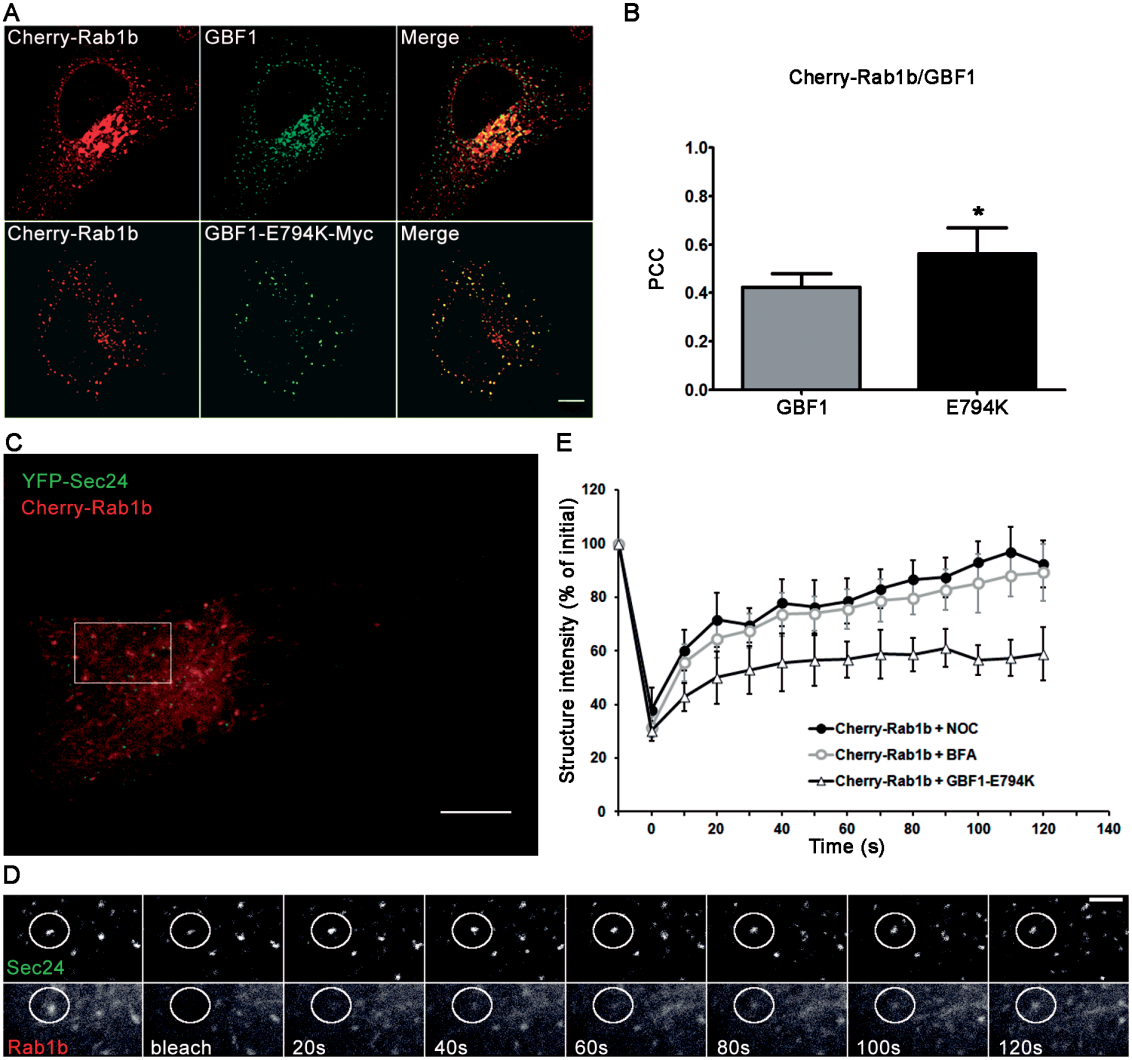
Rab1b and GBF1 co-localization and dynamic analysis. (A and B) Confocal microscopy and co-localization analysis of mCherry-Rab1b and GBF1 or mCherry-Rab1b and GBF1-E794K-myc. Data are expressed as mean ± SD (* = p<0.05). (C-E) Kinetics of mCherry-Rab1b binding to and dissociation from ERES structures in HeLa cells treated with Nocodazole (2,5 μg/mL for 2h at 37°C), BFA (5 μg/mL for 2h) or expressing GBF1-E794K-myc. (C) Representative FRAP experiment in Hela cells, expressing mCherry-Rab1b and YFP-Sec24 and treated with Nocodazole. (D) Magnification of the ROI indicated in C showing a Rab1b-labeled structure selected for photobleaching (white circle, Rab1b panel) that localizes with a YFP-Sec24 labeled structure (white circle, Sec 24 panel). (E) Quantification of the mCherry-Rab1b intensity of a punctate structure localizing with an ERES (labeled with YFP-Sec24) in Nocodazole treated cells (n = 5), cells incubated with BFA (n = 8), or co-transfected with GBF1-E794K (n = 8); error bars, SD. Scale bars represent 10 μm (A and C) and 3 μm (D).

Overall, the findings showing that Rab1b/COPII co-localization time increased in GBF1-E794K transfected cells (Fig 7), together with published data indicating that Rab1b interacts with GBF1 [33], and that GBF1-E794K was stabilized on the membrane relative to a wild-type GBF1 [36], suggest that Rab1b interacted with GBF1-E794K. Consequently, Rab1 was also stabilized in membranes in GBF1-E794K transfected cells. However, if Rab1b was stabilized, its co-localization with GBF1-E794K or GBF wild type should be different. To test this, we quantified co-localization between mCherry-Rab1b with GBF1 (endogenous) or GBF1-E794-myc (Fig 8A). As shown in Fig 8B, the PCC between Rab1b and GBF1-794-myc was slight, but significantly higher than the PCC between Rab1b and the GBF1 (0.56 ± 0.06 vs 0.43 ± 0.05).

Rab1b cycles from the cytosol to membranes (bound to GDP or GTP respectively) and its membrane association/dissociation dynamics on Golgi membranes has been previously analyzed using FRAP assays by our group [33]. However, Rab1b FRAP assays on punctate structures were not performed because their high mobility and/or transience would obstruct measurement of the fluorescence recovery after photobleaching.

To confirm that Rab1b is stabilized on punctate structures in transfected cells, we measured Rab1b membrane association/dissociation dynamics by performing FRAP assays on Rab1b-labeled punctate structures that co-localized with ERES in cells co-transfected with GFP-Rab1b, YFP-Sec24 and GBF1-E794K-myc. As control, FRAP assays were performed on cells treated only with Nocodazole (NOC), a drug that induces microtubule depolymerization and reduces mobility of the VTCs [32]. NOC treatment allowed us to measure fluorescence recovery of Rab1b-punctate structures without perturbing COPI recruitment or anterograde transport, as in the case of BFA or GBF1-E794K. FRAP analysis of mCherry-Rab1b on punctate structures co-localizing with YFP-Sec24 was performed (Fig 8C–8E). The kinetics of mCherry-Rab1b FRAP in the GBF1-E794K expressing cells displayed a slower recovery than that of the control. Furthermore, there was an important difference in the mobile fractions, with approximately 93% of Rab1b appearing mobile in control cells, while only around 56% was mobile in GBF1-E794K expressing cells (compare percentage recovery of Rab1b 100 seconds after photobleaching). We also performed FRAP assays on Rab1b-labeled punctate structures in BFA treated cells, and interestingly, the kinetics of the fluorescence recovery was similar in NOC and BFA treated cells (Fig 8E).

It was puzzling to observe that Rab1b/COPII co-localization time was similar between BFA treated cells and GBF1-E794K expressing cells, while the FRAP assays revealed that Rab1b had different association/dissociation dynamics for these two conditions. However it is known that BFA induces GBF1 redistribution to ER membranes, while GBF1-E794K is distributed in a punctate pattern that co-localizes with ERES and VTCs [8]. Taking into account that Rab1b interacts with GBF1, our FRAP analysis strongly suggests that Rab1b interacts with GBF1-E794K and remains stabilized due to this interaction. The results also indicated that Rab1b association/dissociation dynamics at ERES was dependent on both GBF1 activity and its membrane localization at the ERES-Golgi interface.

## Discussion

In this work, a comprehensive study of Rab1b and its effectors at the ER-Golgi interface was performed under different transport conditions. We showed co-localization of Rab1b with ERES, VTCs and Golgi markers at steady state and after complete Golgi redistribution to the ER induced by BFA treatment (Figs 1 and 2). Although quantitative co-localization information obtained with GFP-Rab1b expressing cells is questionable because it may not reflect the endogenous absolute quantitative values, we emphasize that the most important information derived from our results is the relative Rab1b distribution in structures involved in ER to Golgi transport, in both steady state and BFA treated cells. In this study, we used tagged proteins for two main reasons: First, because it is difficult to find good and compatible antibodies (in terms of animal species) to perform double-labeled immunofluorescence assays, and second, because we wanted to perform all the analysis under comparable conditions, and it was necessary to use tagged proteins to perform the *in vivo* experiments.

At steady state, the highest co-localization of Rab1b was with p115, GM130 and GalNAcT2 (PCC~0.6, 0,61, and 0,53, respectively), with a lower degree of co-localization with ERGIC and Sec31 (PCCs~0.40 and 0.24, respectively). These data agree with results showing that Rab1b interacts with cis-medial Golgi proteins such as p115, GM130, golgin-84, and Giantin [14, 15, 17, 18, 40–42], and with the fact that detection of endogenous Rab1b (using a monoclonal antibody) revealed its Golgi localization [12]. However, this antibody also detected some ER distribution. Thus, we quantified Rab1b co-localization with the ER marker calreticulin, and the PCC was similar to that measured for Sec31 (PCC~0,22, data not shown). We did not detect Rab1b concentration in the pericentrosomal domain of the intermediate compartment as previously described in NRK cells [43], probably because in our system (HeLa cells) this pattern was less evident.

After BFA treatment, Rab1b redistributed to punctate structures in the cytoplasm, and as previously described, ERGIC, p115, and GM130 also distributed to punctate structures [31]. BFA-induced Rab1b punctate structures maintained a high co-localization with p115, but showed less with GM130. In contrast, Rab1b and GalNacT2 co-localization decreased after BFA treatment due to ER relocation of GalNacT2 (PCC changed from 0.53 to 0.39), while the Rab1b and ERGIC53 co-localization did not change after BFA (PCC = 0.39 in both situations). This result is in disagreement with data showing that co-localization of the Rab1a isoform with p58 (the rat homologue of human ERGIC53) increased after BFA treatment [44]. This discrepancy could be due to differential behaviors of the Rab1 isoforms. Moreover, both p115 and Rab1b co-localization with Sec31 increased after BFA treatment, in agreement with Allan *et al*., who postulated that p115 recruits Rab1 onto COPII vesicles [14]. It is puzzling however, that after BFA treatment, Rab1b/p115 co-localization (PCC = 0.60 ± 0.06) was higher than Rab1b/ERGIC53 (PCC = 0.39 ± 0.08). It is possible that the BFA-remnants may be compositionally different and therefore some proteins exhibiting punctate structures (after BFA treatment) are differentially distributed among them. This explanation is supported by previous results showing that not all golgins/GRASPs proteins respond in a similar manner to BFA, and that they differentially distribute in BFA remnants [9]. Accordingly, ERGIC53 and ERGIC32 (ERGIC localized protein) did not exhibit a 100% overlap after BFA treatment [10].

The quantitative Rab1b co-localization with ERES and VTCs performed here will be improved in the future using super resolution microscopy. However, the approach used here was able to detect a higher localization of Rab1b in p115-labeled structures than ERES. The data suggest that even when all cargo exit is blocked at the ER and Rab1b participates in cargo sorting at ERES [24], the bulk of Rab1b activity is required at p115 punctate structures. In agreement, Rab1b, p115 and GM130 interactions are also fundamental in post-ERES events [45].

When the dynamics of mCherry-Rab1b with COPII (labeled with YFP-Sec24) and mCherry-Rab1b with GFP-p115 were visualized simultaneously, the results indicated that Rab1b exhibited a mixed dynamic behavior, with some Rab1b punctate structures behaving like COPII structures (being rather immobile and stable), while others behaved like p115-labeled structures, by describing a trajectory. In agreement with previous analysis performed with the GFP variant of the Rab1a isoform [46, 47], Rab1b also exhibited a tubular-like pattern (Figs 3 and 4, S1 and S2 Movies). These tubular structures were proposed to connect the ERGIC (or intermediate compartment) with the cell periphery or with the endosomal system. In addition, tubules containing Golgi matrix proteins, including p115, were previously described as being involved in Golgi to ER retrograde transport in a protein kinase A and phospholipase D dependent manner [48, 49]. The fact that most of Rab1btubular-like structures localized with p115-labeled tubules agrees with the finding showing that in Golgi, p115 recruits Rab1b [45]. Moreover, punctate Rab1b structures were able to associate to punctate p115 structures in a both stable and transient manner. Interestingly, the appearance or vanishing of some punctate Rab1b, Sec24 and p115-labeled structures occurred independently of each other, suggesting that an association between them was not required to achieve these dynamic behaviors.

Our analysis of Rab1b overlap to long-lived ERES structures during BFA washout (Figs 5 and 6) indicated that all the COPII structures analyzed (50 from 4 cells, from independent washout experiments) associated with Rab1b at some point during the washout. This suggests that during the anterograde transport of cargo, Rab1b was able to associate to all COPII stable structures. The SialT2-CFP sorting/concentration was detected in only 30 of these COPII structures, and during the analyzed time displayed 62 concentration events, suggesting that SialT1-CFP was preferentially sorted in the same group of COPII structures. Consistent with this idea, the ER export of GPI-anchored proteins is restricted to a subpopulation of COPII structures in a Sec24-isoform-selective manner [50]. However, we cannot exclude the possibility that SialT2-CFP might have concentrated in all COPII structures at a different time from the observed period.

Interestingly, an association of SialT2-CFP to COPII structures was mostly detected in Rab1 positive structures, with a spatial-temporal analysis of these SialT2-CFP concentration events indicating that most (~82%) occurred at the same time, or 30 seconds either before or after the Rab1b PFIVar peaks (Fig 6). In agreement, our previous data indicate that Rab1b inhibition delays cargo sorting at ER exit sites [24]. Taken together, the data support the idea that Rab1b modulates COPII sorting by multiple association-dissociation rounds at the COPII structures.

In this study we have characterized, for first time, the effect of GBF1 activity on Rab1b dynamics at the ERES-Golgi interface. Dual expression *in vivo* time-lapse microscopy studies revealed that Rab1b/COPII co-localization is mostly transient. Quantification of the time of association of Rab1b on COPII stable structures indicated that the median co-localization time was ~150 seconds. Interestingly, inhibition of GBF1 activity (by BFA treatment or expression of the dominant GBF1 mutant, GBF1-E794K) significantly modified the transiency of the Rab1b/COPII association, and the co-localization time increased at least 4 times after GBF1 inhibition (Fig 7). At the ERES-Golgi interface, Rab1b recruits GBF1 [33], which is involved in COPI recruitment through the exchange of GDP for GTP on Arf1 [8, 10]. BFA inhibits GBF1 activity, induces Arf1 dissociation from membranes, and therefore blocks COPI recruitment, and it has been reported at the VTCs stage that COPII is exchanged for COPI and the transport of cargo progresses forward. Consequently, without COPI recruitment (due to BFA or GBF1-E794K expression), the advance of transport to the next step was obstructed and therefore Rab1b remained positioned at the ERES structures and increased the time of co-localization to these. Since the degree of Rab1b/Sec24 co-localization was variable, we cannot exclude the possibility that the partial overlap between signals may represent a close proximity between Rab1b and Sec24 labeled structures (visually distinct punctate structures should be separated by at least 200 nm). Furthermore, although GBF1 was inhibited in BFA treated cells, the Rab1b membrane association/dissociation dynamics to ERES structures was not modified by BFA with respect to control cells (Fig 8E). Likewise, BFA did not modify the membrane association/dissociation of the COPII proteins [31]. In contrast, Rab1b association/dissociation kinetics in control or BFA treated cells was significantly different to the GBF1-E794K expressing cells, with the Rab1b mobile fraction decreasing from ~90% to ~55% in GBF1-E794K transfected cells. As Rab1b interacts with GBF1, we also speculate that Rab1b interacts with GBF1-E794K. In agreement with this, Rab1b and GBF1-E794K co-localize in peripheral punctate structures (Fig 8A and 8B) and GBF1-E794K membrane turnover is stabilized relative to the GBF1 wild type [36]. Taken together, these results suggest that, after recruiting GBF1, Rab1b dissociation from membranes is facilitated by the displacement of GBF1 and a regular GDP/GTP exchange on ARF1, and consequently, by COPI recruitment. Summing up, BFA increases the co-localization time of Rab1b to ERES without perturbing the Rab1b membrane association/dissociation dynamics, and therefore without disturbing either the GEF or the GAP of Rab1b. Possibly in BFA-treated cells, Rab1b was unable to interact with GBF1, which is stably associated to the ER, with Rab1b continuing to cycle on and off to ERES membranes, in a state of “waiting” for GBF1. In contrast, in GBF1-E794K expressing cells, Rab1b and GBF1-E794K form a stable association. The Rab1b dynamics of fluorescence recovery in this context was similar to that of the Rab1b-GTP mutant, Rab1b-Q67L [33], suggesting that the Rab1b-GBF1-E794K complex hampers GTP hydrolysis on Rab1b. However, we cannot exclude the possibility that GBF1-E794K inhibits TBC1D20 GAP activity on Rab1b [51]. Our data allows us to postulate that Rab1b coordinates COPII-COPI exchange at the ERES-Golgi interface in a GBF1/COPI regulated manner.

## Supporting Information

S1 Fig Representative immunofluorescence images showing endogenous Rab1b and GM130. Confocal images of HeLa Cells in control (-BFA) and BFA-treated cells (5 ug/mL for 2h).
(TIF)

S1 Movie Representative time-lapse microscopy study of mCherry-Rab1 and YFP-Sec24 expressed in HeLa cells. Images in grayscale show single channels and their co-localization is depicted in the merge channel. Individual images and magnification of the different ROIs and structures indicated with arrows are shown in Fig 3. Images were acquired every 6 seconds over a total time of 7 min (455 sec). The movie was compressed to 10 frames/s.
(MP4)

S2 Movie Representative time-lapse microscopy study of mCherry-Rab1b and GFP-p115 expressed in HeLa cells. Images in grayscale show single channels and their co-localization is depicted in the merge channel. Individual images and magnification of the different ROIs and structures indicated with arrows are shown in Fig 4. Images were acquired every 4 seconds over a total time of 6 minutes (392 seconds). The movie was compressed to 10 frames/s.
(MP4)

S3 Movie Representative dual color time-lapse microscopy study of mCherry-Rab1b and YFP-Sec24 expressed in HeLa cells in three different conditions: Control (-BFA), BFA treated cells (+BFA, 5 μg/mL for 2 h), and in cells co-transfected also with GBF1-E794K-myc. Individual images and magnification of the different ROIs and structures indicated with arrows are shown in Fig 7. Images were acquired every 4–6 seconds over a total time of 300 (-BFA) or 600 seconds (+BFA and GBF1-E794K). The movie was compressed to 10 frames/s.
(MP4)

## References

[1] Appenzeller-Herzog C, Hauri HP. The ER-Golgi intermediate compartment (ERGIC): in search of its identity and function. Journal of cell science. 2006;119(Pt 11):2173–83. 10.1242/jcs.03019 . 16723730

[2] Jensen D, Schekman R. COPII-mediated vesicle formation at a glance. Journal of cell science. 2011;124(Pt 1):1–4. 10.1242/jcs.069773 . 21172817

[3] Stephens DJ, Lin-Marq N, Pagano A, Pepperkok R, Paccaud JP. COPI-coated ER-to-Golgi transport complexes segregate from COPII in close proximity to ER exit sites. Journal of cell science. 2000;113 (Pt 12):2177–85. . 10825291 10.1242/jcs.113.12.2177

[4] Donaldson JG, Cassel D, Kahn RA, Klausner RD. ADP-ribosylation factor, a small GTP-binding protein, is required for binding of the coatomer protein beta-COP to Golgi membranes. Proceedings of the National Academy of Sciences of the United States of America. 1992;89(14):6408–12. 1631136 10.1073/pnas.89.14.6408 PMC49510

[5] Bonifacino JS, Lippincott-Schwartz J. Coat proteins: shaping membrane transport. Nature reviews Molecular cell biology. 2003;4(5):409–14. 10.1038/nrm1099 . 12728274

[6] Cox R, Mason-Gamer RJ, Jackson CL, Segev N. Phylogenetic analysis of Sec7-domain-containing Arf nucleotide exchangers. Molecular biology of the cell. 2004;15(4):1487–505. 10.1091/mbc.E03-06-0443 14742722 PMC379250

[7] Claude A, Zhao BP, Kuziemsky CE, Dahan S, Berger SJ, Yan JP, et al GBF1: A novel Golgi-associated BFA-resistant guanine nucleotide exchange factor that displays specificity for ADP-ribosylation factor 5. The Journal of cell biology. 1999;146(1):71–84. 10402461 PMC2199737

[8] Garcia-Mata R, Szul T, Alvarez C, Sztul E. ADP-ribosylation factor/COPI-dependent events at the endoplasmic reticulum-Golgi interface are regulated by the guanine nucleotide exchange factor GBF1. Molecular biology of the cell. 2003;14(6):2250–61. 10.1091/mbc.E02-11-0730 12808027 PMC194875

[9] Zhao X, Claude A, Chun J, Shields DJ, Presley JF, Melancon P. GBF1, a cis-Golgi and VTCs-localized ARF-GEF, is implicated in ER-to-Golgi protein traffic. Journal of cell science. 2006;119(Pt 18):3743–53. 10.1242/jcs.03173 . 16926190

[10] Kawamoto K, Yoshida Y, Tamaki H, Torii S, Shinotsuka C, Yamashina S, et al GBF1, a guanine nucleotide exchange factor for ADP-ribosylation factors, is localized to the cis-Golgi and involved in membrane association of the COPI coat. Traffic. 2002;3(7):483–95. . 12047556 10.1034/j.1600-0854.2002.30705.x

[11] Tisdale EJ, Bourne JR, Khosravi-Far R, Der CJ, Balch WE. GTP-binding mutants of rab1 and rab2 are potent inhibitors of vesicular transport from the endoplasmic reticulum to the Golgi complex. The Journal of cell biology. 1992;119(4):749–61. 1429835 10.1083/jcb.119.4.749 PMC2289685

[12] Plutner H, Cox AD, Pind S, Khosravi-Far R, Bourne JR, Schwaninger R, et al Rab1b regulates vesicular transport between the endoplasmic reticulum and successive Golgi compartments. The Journal of cell biology. 1991;115(1):31–43. 1918138 10.1083/jcb.115.1.31 PMC2289927

[13] Saraste J, Lahtinen U, Goud B. Localization of the small GTP-binding protein rab1p to early compartments of the secretory pathway. Journal of cell science. 1995;108 (Pt 4):1541–52. . 7615674 10.1242/jcs.108.4.1541

[14] Allan BB, Moyer BD, Balch WE. Rab1 recruitment of p115 into a cis-SNARE complex: programming budding COPII vesicles for fusion. Science. 2000;289(5478):444–8. . 10903204 10.1126/science.289.5478.444

[15] Moyer BD, Allan BB, Balch WE. Rab1 interaction with a GM130 effector complex regulates COPII vesicle cis—Golgi tethering. Traffic. 2001;2(4):268–76. . 11285137 10.1034/j.1600-0854.2001.1o007.x

[16] Weide T, Bayer M, Koster M, Siebrasse JP, Peters R, Barnekow A. The Golgi matrix protein GM130: a specific interacting partner of the small GTPase rab1b. EMBO reports. 2001;2(4):336–41. 10.1093/embo-reports/kve065 11306556 PMC1083862

[17] Rosing M, Ossendorf E, Rak A, Barnekow A. Giantin interacts with both the small GTPase Rab6 and Rab1. Experimental cell research. 2007;313(11):2318–25. 10.1016/j.yexcr.2007.03.031 . 17475246

[18] Satoh A, Wang Y, Malsam J, Beard MB, Warren G. Golgin-84 is a rab1 binding partner involved in Golgi structure. Traffic. 2003;4(3):153–61. 12656988 10.1034/j.1600-0854.2003.00103.x PMC3282115

[19] Sztul E, Lupashin V. Role of vesicle tethering factors in the ER-Golgi membrane traffic. FEBS letters. 2009;583(23):3770–83. 10.1016/j.febslet.2009.10.083 19887069 PMC2788073

[20] Sonnichsen B, Lowe M, Levine T, Jamsa E, Dirac-Svejstrup B, Warren G. A role for giantin in docking COPI vesicles to Golgi membranes. The Journal of cell biology. 1998;140(5):1013–21. 9490716 10.1083/jcb.140.5.1013 PMC2132694

[21] Garcia IA, Martinez HE, Alvarez C. Rab1b regulates COPI and COPII dynamics in mammalian cells. Cellular logistics. 2011;1(4):159–63. 10.4161/cl.1.4.18221 22279615 PMC3265928

[22] Hutagalung AH, Novick PJ. Role of Rab GTPases in membrane traffic and cell physiology. Physiological reviews. 2011;91(1):119–49. 10.1152/physrev.00059.2009 21248164 PMC3710122

[23] Alvarez C, Garcia-Mata R, Brandon E, Sztul E. COPI recruitment is modulated by a Rab1b-dependent mechanism. Molecular biology of the cell. 2003;14(5):2116–27. 10.1091/mbc.E02-09-0625 12802079 PMC165101

[24] Slavin I, Garcia IA, Monetta P, Martinez H, Romero N, Alvarez C. Role of Rab1b in COPII dynamics and function. European journal of cell biology. 2011;90(4):301–11. 10.1016/j.ejcb.2010.10.001 . 21093099

[25] Schweizer A, Fransen JA, Bachi T, Ginsel L, Hauri HP. Identification, by a monoclonal antibody, of a 53-kD protein associated with a tubulo-vesicular compartment at the cis-side of the Golgi apparatus. The Journal of cell biology. 1988;107(5):1643–53. 3182932 10.1083/jcb.107.5.1643 PMC2115344

[26] Kirshner H, Aguet F, Sage D, Unser M. 3-D PSF fitting for fluorescence microscopy: implementation and localization application. Journal of microscopy. 2013;249(1):13–25. 10.1111/j.1365-2818.2012.03675.x . 23126323

[27] Aguet F, Geissbuhler S, Marki I, Lasser T, Unser M. Super-resolution orientation estimation and localization of fluorescent dipoles using 3-D steerable filters. Optics express. 2009;17(8):6829–48. . 19365511 10.1364/oe.17.006829

[28] Dukhovny A, Papadopulos A, Hirschberg K. Quantitative live-cell analysis of microtubule-uncoupled cargo-protein sorting in the ER. Journal of cell science. 2008;121(Pt 6):865–76. 10.1242/jcs.019463 . 18303051

[29] Lippincott-Schwartz J, Yuan LC, Bonifacino JS, Klausner RD. Rapid redistribution of Golgi proteins into the ER in cells treated with brefeldin A: evidence for membrane cycling from Golgi to ER. Cell. 1989;56(5):801–13. . 2647301 10.1016/0092-8674(89)90685-5 PMC7173269

[30] Fujiwara T, Oda K, Yokota S, Takatsuki A, Ikehara Y. Brefeldin A causes disassembly of the Golgi complex and accumulation of secretory proteins in the endoplasmic reticulum. The Journal of biological chemistry. 1988;263(34):18545–52. . 3192548

[31] Ward TH, Polishchuk RS, Caplan S, Hirschberg K, Lippincott-Schwartz J. Maintenance of Golgi structure and function depends on the integrity of ER export. The Journal of cell biology. 2001;155(4):557–70. 10.1083/jcb.200107045 11706049 PMC2198855

[32] Hammond AT, Glick BS. Dynamics of transitional endoplasmic reticulum sites in vertebrate cells. Molecular biology of the cell. 2000;11(9):3013–30. 10982397 10.1091/mbc.11.9.3013 PMC14972

[33] Monetta P, Slavin I, Romero N, Alvarez C. Rab1b interacts with GBF1 and modulates both ARF1 dynamics and COPI association. Molecular biology of the cell. 2007;18(7):2400–10. 10.1091/mbc.E06-11-1005 17429068 PMC1924811

[34] Uliana AS, Giraudo CG, Maccioni HJ. Cytoplasmic tails of SialT2 and GalNAcT impose their respective proximal and distal Golgi localization. Traffic. 2006;7(5):604–12. 10.1111/j.1600-0854.2006.00413.x . 16643282

[35] Giraudo CG, Maccioni HJ. Ganglioside glycosyltransferases organize in distinct multienzyme complexes in CHO-K1 cells. The Journal of biological chemistry. 2003;278(41):40262–71. 10.1074/jbc.M305455200 . 12900410

[36] Szul T, Garcia-Mata R, Brandon E, Shestopal S, Alvarez C, Sztul E. Dissection of membrane dynamics of the ARF-guanine nucleotide exchange factor GBF1. Traffic. 2005;6(5):374–85. 10.1111/j.1600-0854.2005.00282.x . 15813748

[37] Niu TK, Pfeifer AC, Lippincott-Schwartz J, Jackson CL. Dynamics of GBF1, a Brefeldin A-sensitive Arf1 exchange factor at the Golgi. Molecular biology of the cell. 2005;16(3):1213–22. 10.1091/mbc.E04-07-0599 15616190 PMC551486

[38] Spano S, Silletta MG, Colanzi A, Alberti S, Fiucci G, Valente C, et al Molecular cloning and functional characterization of brefeldin A-ADP-ribosylated substrate. A novel protein involved in the maintenance of the Golgi structure. The Journal of biological chemistry. 1999;274(25):17705–10. . 10364211 10.1074/jbc.274.25.17705

[39] Colanzi A, Grimaldi G, Catara G, Valente C, Cericola C, Liberali P, et al Molecular mechanism and functional role of brefeldin A-mediated ADP-ribosylation of CtBP1/BARS. Proceedings of the National Academy of Sciences of the United States of America. 2013;110(24):9794–9. 10.1073/pnas.1222413110 23716697 PMC3683763

[40] Beard M, Satoh A, Shorter J, Warren G. A cryptic Rab1-binding site in the p115 tethering protein. The Journal of biological chemistry. 2005;280(27):25840–8. 10.1074/jbc.M503925200 . 15878873

[41] Diao A, Rahman D, Pappin DJ, Lucocq J, Lowe M. The coiled-coil membrane protein golgin-84 is a novel rab effector required for Golgi ribbon formation. The Journal of cell biology. 2003;160(2):201–12. 10.1083/jcb.200207045 12538640 PMC2172652

[42] Linstedt AD, Hauri HP. Giantin, a novel conserved Golgi membrane protein containing a cytoplasmic domain of at least 350 kDa. Molecular biology of the cell. 1993;4(7):679–93. 7691276 10.1091/mbc.4.7.679 PMC300978

[43] Mochizuki Y, Ohashi R, Kawamura T, Iwanari H, Kodama T, Naito M, et al Phosphatidylinositol 3-phosphatase myotubularin-related protein 6 (MTMR6) is regulated by small GTPase Rab1B in the early secretory and autophagic pathways. The Journal of biological chemistry. 2013;288(2):1009–21. 10.1074/jbc.M112.395087 23188820 PMC3542987

[44] Marie M, Dale HA, Kouprina N, Saraste J. Division of the intermediate compartment at the onset of mitosis provides a mechanism for Golgi inheritance. Journal of cell science. 2012;125(Pt 22):5403–16. 10.1242/jcs.108100 . 22946056

[45] Guo Y, Linstedt AD. Binding of the vesicle docking protein p115 to the GTPase Rab1b regulates membrane recruitment of the COPI vesicle coat. Cellular logistics. 2013;3:e27687 10.4161/cl.27687 25332841 PMC4187009

[46] Sannerud R, Marie M, Nizak C, Dale HA, Pernet-Gallay K, Perez F, et al Rab1 defines a novel pathway connecting the pre-Golgi intermediate compartment with the cell periphery. Molecular biology of the cell. 2006;17(4):1514–26. 10.1091/mbc.E05-08-0792 16421253 PMC1415313

[47] Marie M, Dale HA, Sannerud R, Saraste J. The function of the intermediate compartment in pre-Golgi trafficking involves its stable connection with the centrosome. Molecular biology of the cell. 2009;20(20):4458–70. 10.1091/mbc.E08-12-1229 19710425 PMC2762134

[48] Tenorio MJ, Luchsinger C, Mardones GA. Protein kinase A activity is necessary for fission and fusion of Golgi to endoplasmic reticulum retrograde tubules. PloS one. 2015;10(8):e0135260 10.1371/journal.pone.0135260 26258546 PMC4530959

[49] Martinez-Martinez N, Martinez-Alonso E, Ballesta J, Martinez-Menarguez JA. Phospholipase D2 is involved in the formation of Golgi tubules and ArfGAP1 recruitment. PloS one. 2014;9(10):e111685 10.1371/journal.pone.0111685 25354038 PMC4213061

[50] Bonnon C, Wendeler MW, Paccaud JP, Hauri HP. Selective export of human GPI-anchored proteins from the endoplasmic reticulum. Journal of cell science. 2010;123(Pt 10):1705–15. 10.1242/jcs.062950 . 20427317

[51] Haas AK, Yoshimura S, Stephens DJ, Preisinger C, Fuchs E, Barr FA. Analysis of GTPase-activating proteins: Rab1 and Rab43 are key Rabs required to maintain a functional Golgi complex in human cells. Journal of cell science. 2007;120(Pt 17):2997–3010. 10.1242/jcs.014225 . 17684057

