# Supplementary figures and images for: Spatial-Temporal Study of Rab1b Dynamics and Function at the ER-Golgi Interface
Source: PLoS One. 2016 Aug 8;11(8):e0160838. doi: 10.1371/journal.pone.0160838 (PMC4976911; doi:10.1371/journal.pone.0160838)

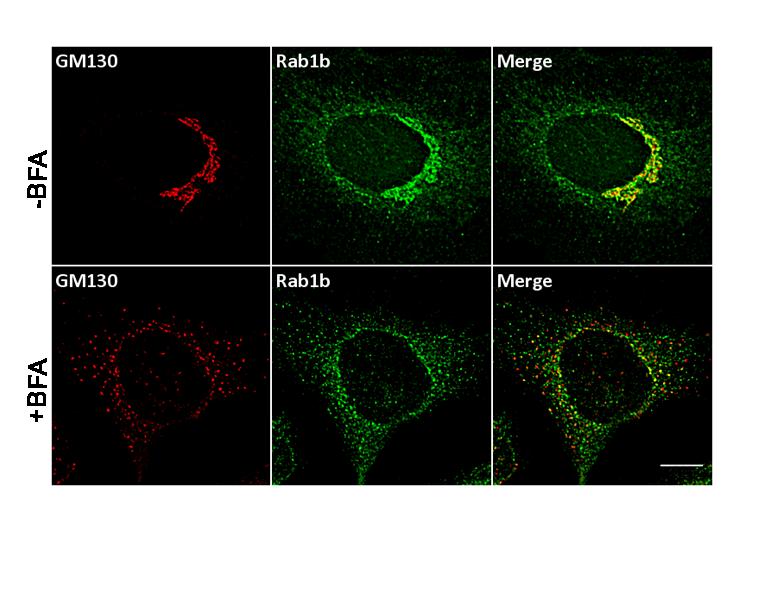

Supplement: S1 Fig — Confocal images of HeLa Cells in control (-BFA) and BFA-treated cells (5 ug/mL for 2h). (TIF) [file pone.0160838.s001.tif]
